# Fast THz-TDS Reflection Imaging with ECOPS—Point-by-Point versus Line-by-Line Scanning

**DOI:** 10.3390/s22228813

**Published:** 2022-11-15

**Authors:** Norbert Pałka, Marcin Maciejewski, Kamil Kamiński, Marek Piszczek, Przemysław Zagrajek, Elżbieta Czerwińska, Michał Walczakowski, Krzysztof Dragan, Piotr Synaszko, Waldemar Świderski

**Affiliations:** 1 Institute of Optoelectronics, Military University of Technology, 2 Kaliski Street, 00-908 Warsaw, Poland; 2 Air Force Institute of Technology, 6 Książe Bolesław Street, 01-494 Warsaw, Poland; 3 Military Institute of Armament Technology, Prymasa Stefana Wyszyńskiego 7 Street, 05-220 Zielonka, Poland

**Keywords:** terahertz imaging, time-domain spectroscopy, nondestructive testing

## Abstract

We built a high-speed TDS setup with the use of electronically controlled optical sampling (ECOPS), which can measure up to 1600 terahertz pulses per second. The movement of the sample was provided by two fast-speed motorized linear stages constituting the gantry. We developed a flat-bar-based metal marker approach for the synchronization of continuous line-by-line scans. We carefully compared the performance of the terahertz reflection time-domain spectroscopy (TDS) scanner operating in a slow point-by-point and a one-hundred-times faster line-by-line imaging scheme. We analyzed images obtained for both schemes for a uniform metallic breadboard with holes, as well as a glass composite sample with defects. Although the measurement time was reduced by 100 times in terms of the line-by-line scheme, the overall performance in both schemes was almost identical in terms of the defects’ sizes, shapes and locations. The results proved that the proposed ECOPS TDS system can provide uniform and extremely fast scanning without any deterioration in image quality.

## 1. Introduction

Time-domain spectroscopy (TDS) is a synchronized emitting-detecting setup [1,2], working in approximately a 0.1–5 THz range. In a classical setup, the optical beam from a femtosecond laser is divided into two parts, one of which is directed to the emitter, while the other passes through the delay line and reaches the receiver. Pulses of electromagnetic radiation (electric field) lasting approximately 1 ps are generated in the emitter. Thanks to the optical system (mirrors and/or lenses), they reach the receiver. A THz pulse is synthetized due to the use of a delay line. The major advantage of this technique is its high signal-to-noise ratio (S/N) in the range up to 90 dB, which diminishes together with the increase in frequency [3].

The TDS method is a widely used technique for the imaging and nondestructive evaluation of the internal structures of many elements and structures [4,5,6], including composite materials, pharmaceuticals, pipes, papers, etc. In TDS scanning systems, an image is created by raster scanning of the samples (point-by-point) both in transmission and reflection configurations. The transmission configuration allows for obtaining only 2D images of the sample, without information about the depth of defects [7]. Much more information about the internal structure of the sample can be obtained from the reflection configuration [8,9,10]. The THz pulse propagating through a multilayer sample is partially reflected from each interface between two media. As a result, the receiver registers the signal, consisting of a series of pulses corresponding to interfaces between media. The distance between these pulses corresponds to the double time of propagation between subsequent layers [11].

Most of the time-domain terahertz systems built to date use mechanical delay systems based on linear stages or rotary elements. However, such mechanical systems represent a major limitation in terms of the speed of measurement. This becomes especially important when large samples are to be evaluated in a short time, where the acquisition frequency of a conventional terahertz system, in the range of several to several dozen Hz, is found to be too slow.

The much faster electronically controlled optical sampling (ECOPS) scheme uses two femtosecond lasers, which eliminates the need for a mechanical delay [12,13]. The terahertz transmitter and receiver are illuminated by separate lasers. To vary the time of arrival of the “read” laser pulses at the receiver, the cavity length—and hence the repetition frequency—of one of the lasers is modulated by a piezoelectric system. For this purpose, the cavity of a master laser has a fixed length, while a slave laser has a short path in free space, where the light is coupled to a fiber optic oscillator and directed at a mirror attached to the piezoelectric system. By means of a fast feedback loop acting on the piezoelectric cavity, the repetition rate of the slave laser is phase-synchronized to the repetition rate of the master laser. This additional sinusoidal modulation changes the phase difference between the two pulse trains, and therefore the pulses from the slave laser are periodically accelerated or delayed. As a result, this system works similarly to a classic TDS system, but the acquisition frequency of the THz signal is up to one hundred times higher.

ECOPS-based TDS systems have already found a number of applications. Molter et al. presented ECOPS-based measurements of sub-wavelength layers in multilayer systems at a rate of 1.6 kHz, where the individual layer thicknesses were analyzed in real time [14]. Fosodeder et al. used ECOPS for fast computed tomography measurements [15]. Yahyapour et al. employed ECOPS for thickness gauging in a piece of an undoped wafer of high-resistivity silicon [13]. Yee et al. demonstrated the imaging of a glass-fiber-reinforced polymer sample with artificial internal defects [16].

In this paper, we demonstrate a high-speed ECOPS-based terahertz time-domain spectroscopy scanner in the reflection configuration. Unlike the slow point-by-point scheme, the scanner acquires signals line-by-line at a high uniform speed, significantly reducing the scanning time. Since the purchased commercial system cannot provide synchronization in the fast mode with the encoder of a fast axis motor, we developed a flat-bar-based metal marker for line synchronization. To evaluate the scanner performance and confirm its proper synchronization, we compared images obtained for point-by-point and line-by-line schemes, both for a uniform metallic breadboard with holes and a glass composite sample with defects. Although the measurement time was reduced by 100 times, overall image performance was almost identical, proving that the proposed ECOPS TDS system can provide extremely fast and uniform scanning.

## 2. ECOPS-Based Scanner

### 2.1. ECOPS Platform

**Figure 1:**
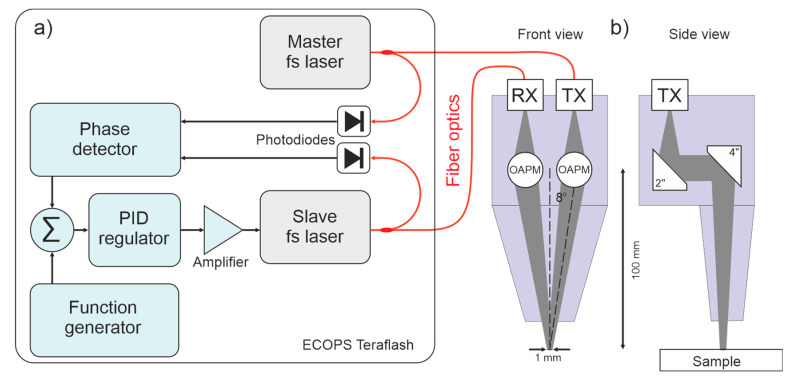
Scheme of Toptica TERAFLASH platform (**a**) and the reflection THz head (**b**) (based on 17).

In our system, we used the ECOPS-based Toptica TERAFLASH platform with a reflection THz head (Figure 1) [17]. The platform uses two 25 mW fiber-based femtosecond lasers (maser and slave) centered at 1560 nm. Their repetition rate is 80 MHz, and the pulse width is typically 80 fs. The repetition rate of each laser is measured by the photodiode and is tuned by adjusting the temperature of the fiber oscillator. The slave laser is stabilized to the clock of the master laser by the phase-locked loop. The 1600 Hz periodic modulation is imposed on the slave laser by the function generator.

Femtosecond pulses are delivered to the InGaAs-based photoconductive switches through the 10-m-long polarization-maintaining fiber optics, which contain a section of specific fiber optics for dispersion compensation. The emitter (TX) with a stripline antenna is based on a high-mobility InAlAs/InGaAs multilayer heterostructure [18,19]. The receiver (RX) with a dipole antenna employs a low-temperature-grown beryllium-doped InAlAs/InGaAs multilayer structure with a short carrier lifetime [20]. The emitted terahertz radiation is linearly polarized.

For the considered samples, the reflection configuration of the emitter-receiver was selected. The THz head consisted of four 2-inch-diameter, off-axis parabolic mirrors that generated a focus on the surface of the sample and, after reflection, directed the beam onto the receiver (Figure 1b). The incident angle was 8 degrees, while the focus had a diameter of approx. 1 mm at 1 THz. The THz head was purged with dry air to remove unwanted influences of water vapor.

### 2.2. Jitter Correction

**Figure 2:**
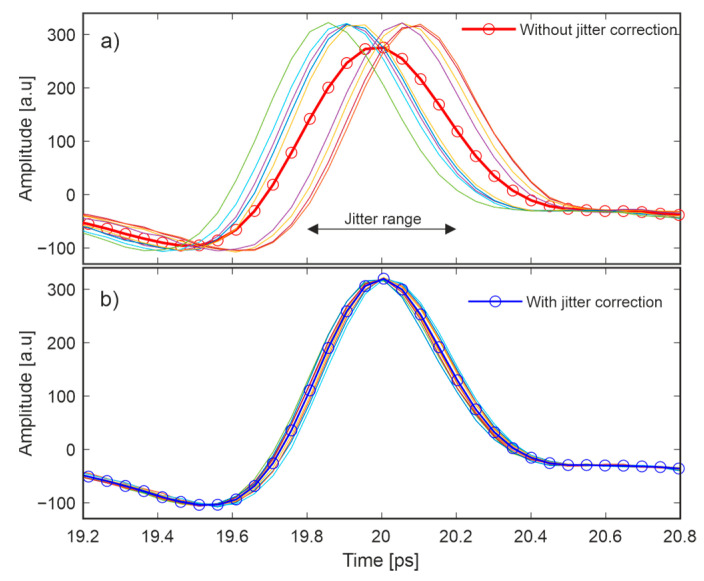
Ten waveforms and their averaging (line with circles) without (**a**) and with (**b**) jitter correction.

The ECOPS systems inherently suffer from instability of the pulse position, called jitter. Figure 2a presents 10 randomly selected pulses from a set of 100 waveforms consecutively measured in 0.0625 s. All waveforms were acquired at one point of the gold mirror, without any movement of the head. Their maxima fluctuate in the range of approximately ±0.2 ps, which deteriorates the measurement quality. The axial resolution of the developed system is about 50 μm in case of the composite sample considered in this work.

Simple averaging resulted in undesirable pulse broadening and a reduction in its amplitude, as shown in Figure 2a. Therefore, a jitter correction scheme to shift the maxima of all pulses to a single point was applied. We analyzed five jitter correction schemes, namely median as well as arithmetic, geometric, harmonic, and trimmed means. After the calculations, it was found that the median scheme gave the best results and was used for further analysis. The pulse averaged over ten jitter-corrected pulses features the same amplitude and time width as the raw measured waveforms (Figure 2b). We note that there is still room for further refinement of the jitter correction scheme, but this requires more computing power and time, which would lead to slower scanning, and therefore it has not been implemented.

### 2.3. Parameters of the Waveforms

**Figure 3:**
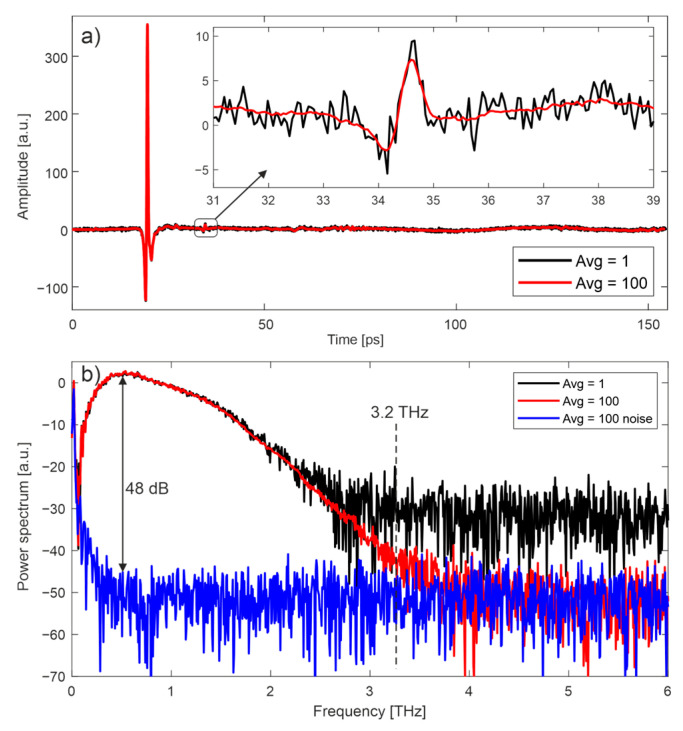
THz waveforms for averaging 1 and 100 (**a**) and their power spectra (**b**). Inset shows influence of averaging on the smoothing of the signal.

Figure 3 presents terahertz waveforms and their spectra measured with jitter correction for two averaging values considered in this work: 1 (no averaging) and 100. The radiation was reflected from a gold mirror placed in the focus of the THz beam. Each waveform was approximately 154 ps long and its main pulse lasted around 0.35 ps (FWHM). The inset in Figure 3a shows the influence of averaging on the smoothing of the signal. The dynamic range [21] and the spectrum range of a single waveform were approximately 25 dB and 2.2 THz, respectively, and increased to 48 dB and 3.2 THz, respectively, when 100 waveforms were averaged.

### 2.4. Gantry System

**Figure 4:**
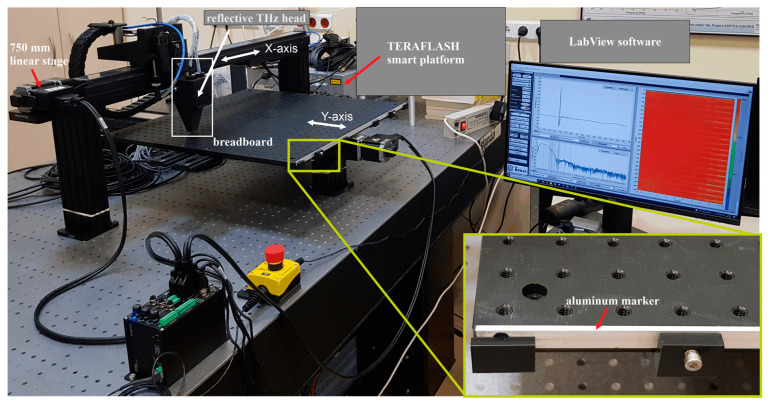
Photo of the ECOPS TDS system.

We built the gantry system for the two-dimensional raster scanning of samples (Figure 4). The system consisted of two perpendicularly installed, motorized lead-screw-driven 750 mm travel linear stages (LRT0750DL-E08CT3A) from the Zaber company, which provided a maximum speed of 700 mm/s, maximum thrust of 200 N, unidirectional accuracy of 0.2 mm, and repeatability equal to 8 μm.

The X-axis stage had the optical head mounted, while the Y-axis stage was equipped with the aluminum breadboard (Thorlabs MB6060/M) with dimensions of 600 × 600 mm. The breadboard has M6 mounting holes every 25 mm. The system can scan a few centimeter-thick samples with size up to 500 × 500 mm. The slow X-axis stage moves the head over the sample point-by-point, while the fast Y-axis stage moves the sample under the THz head point-by-point or line-by-line. This approach minimizes the impact of continuous acceleration and deacceleration of the head, which could adversely affect its operation. The step size of the motor in the X-axis can be freely set with a resolution up to 2 µm.

The software for the scanner was prepared in the LabView 2021 environment. The program architecture was developed in accordance with the Queued Message Handler design pattern, with a separate loop for THz data reading. The result was an application with a responsive user interface protected against data loss. Several main functional blocks can be distinguished in the program, namely for operating Zaber motors, running the Toptica TDS unit, data analysis, and refreshing the user interface. The first two elements were developed based on manufacturers’ libraries—respectively, Zaber A Series.lvlib for engines and DeCOF.lvlib for the TDS unit. The authors entirely prepared the remaining subroutines. The program clearly separates individual functional blocks, which facilitated its development. The gantry can work in two modes: point-by-point or line-by-line. These are described below.

### 2.5. Point-by-Point Scanning Scheme

In this approach, the head was placed over a point of the sample and stopped, the TDS signal was measured, and the head moved to the next point. When the head reaches the end of the sample, it returns to the starting point without taking the measurement. In this scheme, the return time (~0.5 s) is negligible in comparison to the scanning time of the 500 mm line, which was 100–200 s (see Table 1). The scanning was performed at the maximum speed (700 mm/s) and moderate acceleration (2000 mm/s^2^) of the Y-axis motor. Due to use of the encoders with both motors and stopping them for the time of data acquisition, this scheme is accurate and provides an averaging option with the jitter correction for data from one point, which enhances the S/N ratio of the TDS signals. However, it suffers from a long measurement time, because, for each step, the motor should be accelerated and deaccelerated.

### 2.6. Line-by-Line Scanning Scheme

The line-by-line scanning scheme was developed to speed up the scanning process. The Y-axis motor performs a fast, uniform motion without stopping along the entire length of the sample, at the maximum speed of the linear stage (up to 700 mm/s). When the head reaches the end of the sample, it returns to the starting point by taking the measurement. Due to the inertia of the resulting structure, its acceleration along the Y-axis was limited to 5000 mm/s^2^. The scanning time of the 500-mm-long line of sample lasted around 1.4 s.

During this movement, the THz head acquires the signals uniformly at the maximum rate offered by the TDS setup (1600 waveforms/s). In this mode, the maximum resolution is 0.44 mm (700 m/s divided by 1600 waveforms/s). When the line is scanned, the X-axis stage moves the head by the set step and the Y-axis scan begins. In this scheme, there is also the possibility of averaging; however, not a single point but a part of the line is averaged. During averaging, the same jitter correction is used.

**Figure 5:**
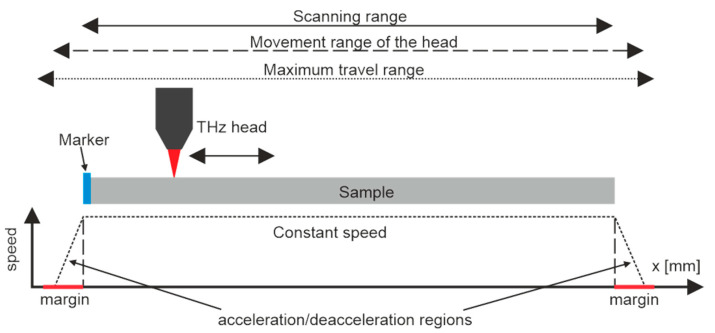
Concept of line-by-line scanning with the synchronization marker.

The challenge in fast continuous linear scanning is synchronization, which can be reduced to the problem of assigning a specific TDS waveform to the proper fragment (point) of the scanned sample. In the considered TeraFlash platform, successive TDS waveforms were recorded with a fixed interval of time. However, it was difficult to determine the beginning and end of a series of waveforms. This problem was solved by placing a 3-mm-wide aluminum flat bar on the edge of the breadboard, which acted as a marker (Figure 5, inset in Figure 4). When scanning, it was always slightly above the sample, so that its waveforms could be easily distinguished from other waveforms related to the sample. The collected series of waveforms were shifted so that the waveform from the synchronization marker provided a clear time stamp. The movement of the THz head was performed in a range wider than the size of the scanned sample. The margins were set so that the THz head could achieve a constant speed during the scanning (Figure 5).

**Figure 6:**
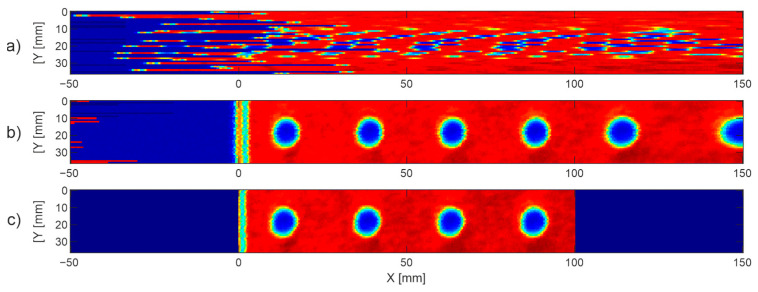
Line shifting algorithm. C-scan of randomly shifted lines (**a**), lines shifted to 0 point (**b**), and resultant image (**c**).

Figure 6 shows the line shifting algorithm for the 40 × 100 mm scanning range of the plate with M6 holes. At the first step, we uniformly scanned the region with 50 mm margins on both sides (movement range of the head), which resulted in the randomly shifted lines (presented as C-scan) shown in Figure 6a. The algorithm then found the unique marker signal in each line and shifted the lines so that their marker signals’ centers matched point 0; see Figure 6b. The first four holes (blue spots) were uniform, while the two last holes were stretched because they were in the margin region, where the speed was decreasing. Finally, the margin regions were removed to show only the image of the sample in the given scanning range (Figure 6c).

In the presented setup, for samples up to 5 kg, the maximum speed is 700 mm/s. For heavier samples, it is necessary to reduce the acceleration in order not to exceed the maximum thrust of the stage. Lower acceleration increases the acceleration distance, which must not be longer than the margin defined as the distance from the marker to the end of the motor’s travel range. In this case, it is necessary to limit the maximum speed so that the acceleration distance remains shorter than the margin. For 15 and 30 kg samples, the maximum speed is around 630 and 570 mm/s, respectively. For the given sample weight, the software automatically calculates the margin, the scanning speed, and the acceleration.

## 3. Verification of the Synchronization

To evaluate the scanner and confirm the proper synchronization of the acquired waveforms, we measured the 12.7-mm-thick aluminum breadboard with M6 threaded holes drilled every 25 mm. Such a plate provides a regular, high-contrast THz image, suitable for verifying the correct operation of the scanner. We measured a 35 × 500 mm area with two rows of holes in both the point-by-point and line-by-line scanning schemes. The step size in both axes in both schemes was 0.44 mm, which provided the same resolution of the resultant images. The sample was measured without averaging and with averaging equal to 100. Table 1 shows the measurement times for the entire sample and a single 500-mm-long line. In the case without averaging, scanning in the line-by-line scheme is roughly 100 times faster in comparison to the point-by-point scheme and only 2.3 times faster for 100 averaging.

**Figure 7:**
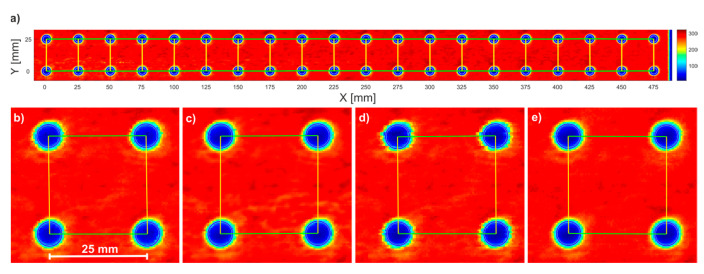
The C-scan of the breadboard for point-by-point without averaging scheme (**a**) and C-scans of first four spots for following schemes: point-by-point without averaging (**b**), point-by-point averaging 100 (**c**), line-by-line without averaging (**d**), and line-by-line averaging 100 (**e**).

Figure 7 presents a C-scan of the sample for the point-by-point without averaging scheme, presented as the peak-to-peak of the THz pulse. One can notice a regular pattern of blue spots corresponding to the M6 holes. C-scans obtained for the remaining three schemes were very similar and hence we present their images limited to the first four spots.

**Figure 8:**
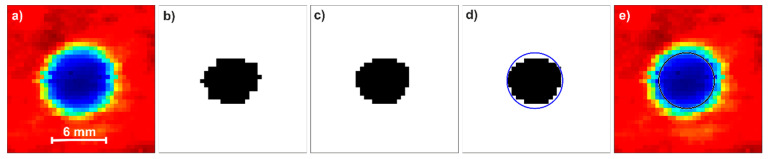
C-scan of the 6 mm hole during processing: raw (**a**), binarization (**b**), filtration (**c**), finding the circle with circular Hough transform (**d**), and the circle marked on the original image (**e**).

To compare the C-scans, we quantified the uniformity of spot distribution. For each spot, we determined a circle with a diameter of 6 mm, whose center was calculated by the following MATLAB-based algorithm (Figure 8). The spots were first binarized using the Otsu method to calculate the global threshold, then filtered by a 2D median filter, and finally the circular Hough transform was applied to find the centers of the circles. The circle diameter is smaller than the size of the hole, which is connected to the THz beam diameter.

In the next step, the distances between the centers of adjacent circles were calculated and averaged independently for the horizontal and vertical axes (Figure 7b–e). Finally, we also determined the distances between the extreme points for the top and bottom rows of points (Table 2). Values obtained for all schemes are very similar and agree well with the real values (25 mm and 475 mm).

**Figure 9:**
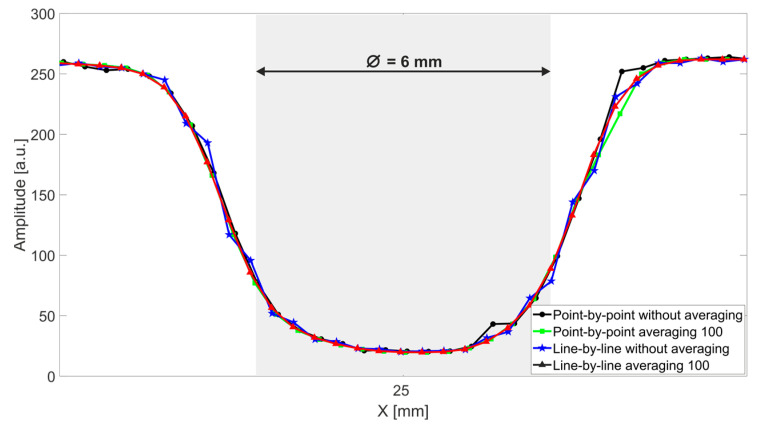
Peak-to-peak amplitude of the terahertz signal reflected along the line passing through the M6 hole.

For an even more detailed comparison of the four scanning schemes under consideration, the peak-to-peak amplitude of the terahertz signal reflected along the line passing through an arbitrarily selected M6 hole is shown (Figure 9).

We can conclude that despite a significant increase in the scanning speed, both the images and averaged distances are similar, which proves the correctness of the synchronization in the line-by-line scheme, as well as the stable and repeatable operation of the motors used. The proposed approach can therefore ensure extremely fast and uniform scanning with the ECOPS TDS system.

## 4. Scanning of the Glass Composite Sample

**Figure 10:**
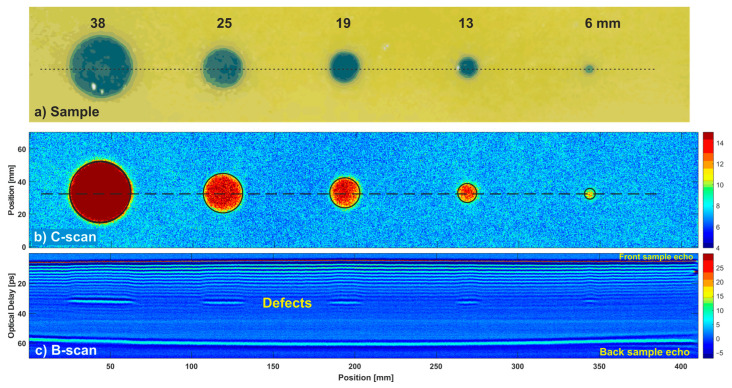
The glass-fiber composite sample with circular defects: photo (**a**), C-scan (**b**) and B-scan (**c**) of the defects. Line-by-line scheme without averaging.

To compare both scanning schemes in a scenario with a real sample, we measured a 160 × 500 mm region of a 3-mm-thick glass-fiber composite plate consisting of 24 layers (Figure 10a). The circular defects, made of a Teflon foil, were situated in the middle of the sample at the depth of around 1.5 mm, between the 12th and 13th layers. We measured the sample both in the point-by-point and line-by-line scanning schemes, without averaging and with averaging equal to 100. The step size in both axes in both schemes was 0.44 mm, which provided the same resolution of the resultant images.

**Figure 11:**
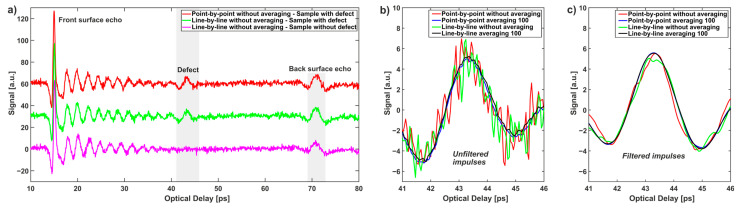
Waveforms reflected from the center of the defect with the diameter of 19 mm (**a**). Unfiltered (**b**) and filtered (**c**) impulses corresponding to the defect.

Figure 10b presents a C-scan of the sample measured in the line-by-line approach (without averaging), calculated as peak-to-peak in the time slice. The time slice range 41–46 ps (depicted in Figure 11a) was selected arbitrarily to optimally show the defects. Each defect was marked with a circle, whose radius was taken from the real defect (Figure 10a), while the center was determined with the circular Hough transform described in Section 3. Figure 10c shows a B-scan presented for the line passing through the centers of the circles (see Figure 10b). In both figures, all defects are clearly seen. The visibility can be improved by the filtering of the signals in the time domain.

Figure 11a shows the waveform reflected from the center of the defect with a diameter of 19 mm, which was selected for further, more detailed analysis. One can notice front and back surface echoes, as well as the pulse reflected from the defect. Moreover, waning oscillations after the front surface echo are connected with reflections from the internal interfaces of glass-fiber layers. They are also visible in Figure 10c.

Figure 11b and c show the magnification of the impulse related to the defect in the four acquisition modes under consideration: raw and after filtering with a band pass filter with a low frequency of 0.1 THz and a high frequency of 1 THz, respectively. The noise level of both raw signals is significantly but similarly high, but, after filtration, all pulses are consistent in relation to their shape, amplitude, and duration.

**Figure 12:**
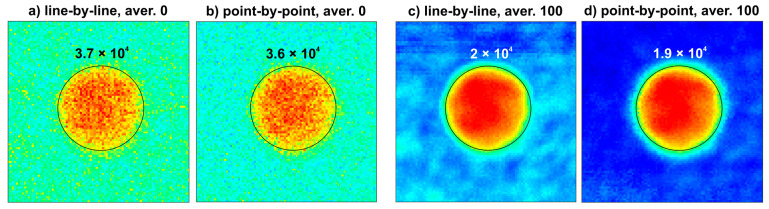
C-scans of the 19-mm-diameter defect area measured with various schemes. All images have the same resolution.

Finally, Figure 12 presents C-scans (peak-to-peak in the time slice mode) for the area with the defect with the diameter of 19 mm obtained for four acquisition modes. For a qualitative assessment, we calculated the sum of all pixels inside the circles. The obtained values differed by around 3–5% between corresponding images, which proves their good similarity.

## 5. Conclusions

The paper describes the TDS ECOPS technique with the acquisition of 1600 signals per second. Such high-speed measurement inherently suffers from the jitter of the pulse position, for which a correction method was developed. Then, time signals and their spectra were presented for two averaging values considered in this paper: 1 and 100. The measurement dynamics are lower than in the classic TDS system with a delay line. Afterwards, the high-speed THz scanner based on ECOPS for the reflection imaging of samples was presented. We next described in detail the gantry motors, LabView-based software for scanner operation, and two image acquisition schemes, namely point-by-point and line-by-line. Due to the lack of electronic position synchronization, we developed the optical synchronization with the flat-bar-based metal marker and the line shifting algorithm.

In order to evaluate the scanner performance, we measured two samples—a uniform metallic breadboard with M6 holes and a glass composite sample with defects—both in the point-by-point and line-by-line scheme. The measurement results of the first sample proved the correctness of the synchronization in the line-by-line scheme, as well as the stable and repeatable operation of the motors used. We also measured the glass composite sample with Teflon-based defects. In this case, time-domain signals and images were very similar for both acquisition schemes, regardless of the averaging.

We can conclude that although the measurement time was reduced by a factor of 100 in the line-by-line scheme, the overall performance of the two acquisition schemes was nearly identical in size, shape, and defect location. The results proved that the proposed ECOPS TDS system with the line-by-line scheme can provide uniform and extremely fast scanning of samples in the reflection configuration.

## Figures and Tables

**Table 1:** Measurement times for different scanning schemes.

Scanning Scheme	Measurement Time Total [min.]	Measurement Time per Line [s.]
Point-by-point without averaging	208.9	136.3
Point-by-point averaging 100	306.6	199.7
Line-by-line without averaging	2.2	1.4
Line-by-line averaging 100	128.2	83.6

**Table 2:** Horizontal and vertical means for different scanning schemes.

	Mean Distances between the Centers of Adjacent Circles		Distances between the Extreme Points	
Scanning Scheme	Horizontal [mm]	Vertical [mm]	Top Line [mm]	Bottom Line [mm]
Point-by-point without averaging	25.01 ± 0.11	25.07 ± 0.17	475.19	475.10
Point-by-point averaging 100	25.01 ± 0.09	25.02 ± 0.12	475.04	475.20
Line-by-line without averaging	25.00 ± 0.12	25.06 ± 0.14	475.07	474.98
Line-by-line averaging 100	25.00 ± 0.10	25.00 ± 0.11	475.10	475.03

## Data Availability

Not applicable.

## References

[1] Neu J., Schmuttenmaer C.A. (2018). Tutorial: An Introduction to Terahertz Time Domain Spectroscopy (THz-TDS). J. Appl. Phys..

[2] Hangyo M., Tani M., Nagashima T. (2005). Terahertz Time-Domain Spectroscopy of Solids: A Review. Int. J. Infrared Millim. Waves.

[3] Vieweg N., Rettich F., Deninger A., Roehle H., Dietz R., Gobel T. A Time-Domain Terahertz Spectrometer with 90 DB Dynamic Range. Proceedings of the International Conference on Infrared, Millimeter, and Terahertz Waves, IRMMW-THz.

[4] Castro-Camus E., Koch M., Mittleman D.M. (2021). Recent Advances in Terahertz Imaging: 1999 to 2021. Appl. Phys. B.

[5] Valušis G., Lisauskas A., Yuan H., Knap W., Roskos H.G. (2021). Roadmap of Terahertz Imaging 2021. Sensors.

[6] Stoik C., Bohn M., Blackshire J. (2010). Nondestructive Evaluation of Aircraft Composites Using Reflective Terahertz Time Domain Spectroscopy. NDT E Int..

[7] Hu B.B., Nuss M.C. (1995). Imaging with Terahertz Waves. Opt. Lett..

[8] Palka N., Miedzinska D. (2014). Detailed Non-Destructive Evaluation of UHMWPE Composites in the Terahertz Range. Opt. Quantum Electron..

[9] Ellrich F., Bauer M., Schreiner N., Keil A., Pfeiffer T., Klier J., Weber S., Jonuscheit J., Friederich F., Molter D. (2020). Terahertz Quality Inspection for Automotive and Aviation Industries. J. Infrared Millim. Terahertz Waves.

[10] Ospald F., Zouaghi W., Beigang R., Matheis C., Jonuscheit J., Recur B., Guillet J.-P., Mounaix P., Vleugels W., Bosom P.V. (2013). Aeronautics Composite Material Inspection with a Terahertz Time-Domain Spectroscopy System. Opt. Eng..

[11] Palka N., Krimi S., Ospald F., Miedzinska D., Gieleta R., Malek M., Beigang R. (2015). Precise Determination of Thicknesses of Multilayer Polyethylene Composite Materials by Terahertz Time-Domain Spectroscopy. J. Infrared Millim. Terahertz Waves.

[12] Yee D.-S., Kim Y. (2010). High-Speed Terahertz Time-Domain Spectroscopy Based on Electronically Controlled Optical Sampling. Opt. Lett..

[13] Yahyapour M., Jahn A., Dutzi K., Puppe T., Leisching P., Schmauss B., Vieweg N., Deninger A. (2019). Fastest Thickness Measurements with a Terahertz Time-Domain System Based on Electronically Controlled Optical Sampling. Appl. Sci..

[14] Molter D., Ellenberger K.S., Klier J., Duran S., Jonuscheit J., von Freymann G., Vieweg N., Deninger A. (2022). Kilohertz Pixel-Rate Multilayer Terahertz Imaging of Subwavelength Coatings. Appl. Sci..

[15] Fosodeder P., Hubmer S., Ploier A., Ploier A., Ramlau R., Ramlau R., van Frank S., Rankl C. (2021). Phase-Contrast THz-CT for Non-Destructive Testing. Opt. Express.

[16] Kim C.Y., Yee D.-S., Yang H.-S., Yahng J.S., Ye J.C., Jin K.H. (2015). High-Speed Terahertz Reflection Three-Dimensional Imaging Using Beam Steering. Opt. Express.

[17] TeraFlash Smart. TOPTICA Photonics AG. https://www.toptica.com/products/terahertz-systems/time-domain/teraflash-smart.

[18] Dietz R.J.B., Globisch B., Gerhard M., Velauthapillai A., Stanze D., Roehle H., Koch M., Göbel T., Schell M. (2013). 64 ΜW Pulsed Terahertz Emission from Growth Optimized InGaAs/InAlAs Heterostructures with Separated Photoconductive and Trapping Regions. Appl. Phys. Lett..

[19] Sartorius B., Stanze D., Gerhard M., Koch M., Schell M., Dietz R.J.B. (2011). THz Generation at 1.55 Μm Excitation: Six-Fold Increase in THz Conversion Efficiency by Separated Photoconductive and Trapping Regions. Opt. Express.

[20] Globisch B., Dietz R.J.B., Stanze D., Göbel T., Schell M. (2014). Carrier Dynamics in Beryllium Doped Low-Temperature-Grown InGaAs/InAlAs. Appl. Phys. Lett..

[21] Naftaly M., Dudley R. (2009). Methodologies for Determining the Dynamic Ranges and Signal-to-Noise Ratios of Terahertz Time-Domain Spectrometers. Opt. Lett..

